# The imperative for culturally specific suicide prevention models beyond the Western gaze

**DOI:** 10.3389/fpsyt.2026.1755211

**Published:** 2026-01-28

**Authors:** Qian-Nan Ruan, Jing Qian, Xiu-Shuang Lin

**Affiliations:** 1 Wenzhou Seventh People’s Hospital, Wenzhou, China; 2 Third Affiliated Hospital of Wenzhou Medical University, Wenzhou, China

**Keywords:** collectivism, cultural specificity, help-seeking behavior, shame-honor, suicide prevention, WEIRD psychology

## Abstract

Suicide remains a critical global public health challenge, with distinct prevalence patterns and risk factors observed across different cultural contexts. While evidence-based prevention strategies, such as Cognitive Behavioral Therapy (CBT), crisis hotlines, and gatekeeper training, have been developed predominantly in Western settings and have demonstrated efficacy, their application in non-Western populations faces unique cultural hurdles. This perspective article examines the limitations of applying individualistic suicide prevention models without sufficient adaptation to East Asian cultural contexts, which are often characterized by “shame-honor” dynamics and collectivist values. We acknowledge the significant contributions of existing Western frameworks but argue that a reliance on these models, if unexamined, may overlook specific barriers to help-seeking, such as the intense stigma associated with “loss of face” and the perception of mental distress as a burden to the family. By analyzing the “cultural mismatch” between the Western independent self and the East Asian interdependent self, this article calls for a paradigm shift toward “cultural grounding.” We propose that the field must move beyond superficial adaptation toward the co-creation of interventions that integrate indigenous cultural narratives and social structures. This approach aims not to replace existing evidence-based practices but to enhance their relevance and accessibility, ensuring that suicide prevention strategies are truly responsive to the diverse realities of global populations.

## 1 Introduction

Suicide represents one of the most pressing public health crises of our time, claiming over 700,000 lives annually (1). It is a global phenomenon that transcends borders, age groups, and socioeconomic strata, necessitating robust prevention strategies worldwide. However, a significant disparity exists in the global mental health landscape: while 77% of global suicides occur in low- and middle-income countries, the theoretical frameworks, assessment tools, and evidence-based interventions designed to prevent them are overwhelmingly products of high-income, Western societies (2, 3). This discrepancy raises important questions about the cross-cultural efficacy of standard prevention models when applied to populations with vastly different social ontologies.

The East Asian region, which bears a heavy suicide burden, serves as a critical context for examining these challenges (4). Despite the implementation of standard prevention toolkits, largely comprising gatekeeper training (5), crisis hotlines (6), and psychotherapies like Cognitive-Behavioral Therapy (CBT) (7), suicide rates in the region remain a significant concern. While these Western-derived interventions have saved countless lives and are utilized widely, their effectiveness can be hampered by deep-seated cultural barriers regarding stigma and communication. We acknowledge that significant strides have been made in the cultural adaptation of Western frameworks. Recent meta-analyses indicate that culturally adapted CBT and DBT have demonstrated efficacy in reducing suicide attempts in various Asian cohorts (8). Current adaptations often involve linguistic translation, the inclusion of metaphors relevant to local contexts, and the matching of therapists’ ethnicity (9). While these adaptations effectively address the delivery and content of care, we posit that they may not fully resolve the deeper fundamental divergence regarding how the self is defined in relation to suffering.

This article seeks to bridge the gap between global evidence-based practices and local cultural realities. We analyze how the “Western-centric” paradigm, while scientifically robust in its context of origin, may face limitations when addressing the “shame-honor” dynamics and collectivist values prevalent in East Asia. Rather than dismissing existing models, we argue for the necessity of evolving them. We propose a move toward culturally grounded approaches that respect local understandings of the self and suffering. This pursuit is not merely a matter of improving clinical efficacy but is fundamentally an ethical imperative to ensure that suicide prevention efforts are accessible and relevant to all populations.

## 2 Examining the cultural assumptions underlying Western suicide prevention models

The dominant approaches to suicide prevention, while grounded in decades of rigorous empirical research, rest upon specific, culturally situated understandings of selfhood, suffering, and healing. These frameworks, developed primarily in Western, Educated, Industrialized, Rich, Democratic (WEIRD) societies, utilize epistemological baselines that may not translate seamlessly across cultural boundaries. As scholars within Western academia increasingly critique the universality of psychological theories derived from this narrow population base (10), it becomes imperative to examine how these assumptions function, or potentially dysfunction, in East Asian contexts.

The first foundational assumption is individualization. Western psychology typically conceptualizes the self as a discrete, autonomous, and bounded entity, with psychological processes occurring primarily within the independent individual (11). Consequently, intervention efforts are directed toward individual-level change, specifically modifying internal cognition, neurobiology, or behavior. While generative for treatment development, this framework may inadequately capture the experience of the interdependent self, where identity is fundamentally relational. In contexts where the self is defined by porous boundaries and shared fate with the family, locating the “problem” solely within the individual mind can feel culturally incongruent.

Closely related is the framing of pathology. While contemporary psychiatry operates within a biopsychosocial model that officially recognizes social and environmental contexts, the practical application often leans heavily on a biomedical core. In this framework, suicide is predominantly conceptualized as a downstream consequence of diagnosable mental disorders (12), potentially overshadowing the sociomoral triggers that are central in collective societies. It is well-established that approximately 90% of suicide cases globally are associated with diagnosable mental disorders (13). We do not dispute this critical clinical reality. However, strict reliance on psychiatric diagnosis may overlook the high prevalence of impulsive suicide in East Asia, where individuals without a prior history of mental illness may act fatally in response to acute psychosocial stressors (e.g., academic failure or family conflict) (14). Furthermore, due to stigma and limited mental health resources, many underlying disorders remain under-diagnosed. However, in collective societies, the precipitating triggers and the phenomenology of these disorders often differ. An exclusive focus on the downstream psychopathology may obscure the upstream culturally specific risk factors, such as acute shame or family conflict, that ignite the disorder in the first place (15, 16). In many East Asian contexts, suicide may be precipitated by acute “loss of face,” economic failure, or the inability to fulfill filial duties. These experiences represent profound moral crises rather than merely symptoms of internal brain pathology. By medicalizing these forms of distress, standard prevention models risk invalidating the socio-moral reality of the patient’s suffering.

A third assumption concerns the logocentrism and direct verbal expression. Western therapeutic approaches, including crisis helplines and psychotherapy, place extraordinary emphasis on verbalization as the primary vehicle for healing. The “talking cure” assumes that articulating inner turmoil is inherently therapeutic and that explicit disclosure of suicidal intent is a necessary precursor to help (17). However, this reflects low-context communication norms. In cultures characterized by high-context communication and the preservation of interpersonal harmony, direct verbal disclosure of suicidal ideation may be viewed as disruptive or immature (18). The expectation of explicit verbalization can create a barrier for individuals who are culturally conditioned to express distress through somatic complaints or indirect relational signaling (16).

These assumptions crystallize in influential theoretical frameworks, such as the Interpersonal-Psychological Theory of Suicide (IPTS). Joiner’s theory posits that “perceived burdensomeness” is a proximal cause of suicidal desire (19). In Western clinical application, a client’s belief that “my family would be better off without me” is typically categorized as a cognitive distortion, a depression-induced error in thinking that must be challenged and restructured (20).

However, applying this logic without adaptation to East Asian contexts reveals a “category error (21).” In collectivist cultures, where role obligations and reciprocity are paramount, a failure to contribute to the family unit or the accrual of debt that impacts the extended family constitutes an objective breach of the social contract. In this context, “burdensomeness” may not be a cognitive distortion, but a culturally grounded moral evaluation of one’s failure to fulfill interdependent roles (22). While skilled CBT practitioners may navigate this nuance, standard protocols that categorize this moral reality solely as an ‘irrational thought’ to be corrected, without acknowledging its cultural validity, risk invalidating the patient’s value system, potentially severing the therapeutic alliance. The friction lies not in the theory’s identification of burdensomeness as a risk factor, but in the therapeutic assumption that the sensation of burden is always an internal pathology rather than an interpersonal reality.

## 3 Cultural structures shaping suicide risk in East Asian contexts

The East Asian cultural landscape, profoundly influenced by Confucian, Taoist, and Buddhist philosophical traditions, presents a distinct etiology of suicide risk. However, it is critical to avoid essentializing these cultures as monolithic (23); the region is characterized by rapid modernization, creating a unique friction between traditional collectivist values and contemporary hyper-competitive individualism (24). Within this complex milieu, specific cultural structures interact with psychological distress to create patterns of risk and help-seeking that diverge significantly from Western norms.

### 3.1 The moral anatomy of shame and face

A foundational element distinguishing this context is the primacy of shame (*chi*) over guilt as a regulatory moral emotion. While Western models often operate on a guilt-based logic, where distress arises from specific transgressions that can be absolved through confession and reparation, East Asian contexts are frequently governed by shame, an ontological experience of the self as fundamentally flawed in the eyes of others (25). This dynamic is inextricably linked to *mianzi* (face), which functions not merely as vanity but as collective social capital shared by the family unit.

In this “moral economy,” psychological distress is often interpreted as a failure of self-regulation that threatens collective family honor (26). Consequently, the act of seeking professional help presents a paradox: the intervention designed to alleviate distress (disclosing private family matters to an ‘outsider,’ regardless of professional confidentiality) violates the cultural imperative of *jia chou bu ke wai yang* (family ugliness should not be aired outwardly). This creates a “cycle of silence,” where the shame of experiencing distress is compounded by the shame of needing help, driving suicidal ideation underground where it is shielded from detection by standard screening tools (27).

### 3.2 The double-edged sword of interdependence

The East Asian concept of the interdependent self acts as a double-edged sword regarding suicide risk (28). On one side, the relational definition of self, where one’s value is derived from fulfilling roles as a child, student, or employee, can amplify risk. When an individual perceives themselves as failing in these roles, the Western concept of “perceived burdensomeness” transforms into a grim sociocultural reality. In extreme cases, this aligns with a distorted form of Durkheim’s “altruistic suicide,” where ending one’s life is tragically misconstrued as a moral act to relieve the family of a financial or emotional burden.

Conversely, this same interdependence serves as a potent protective factor. The moral obligation to others often functions as the primary barrier against acting on suicidal urges. Many individuals in East Asian contexts report that the fear of inflicting trauma on parents or children is the sole reason they survive (29). However, for younger generations, this creates a “double bind”: they are caught between the crushing weight of modern economic competition (“involution”) and the traditional imperative to succeed for the family’s sake. This generational dissonance constitutes a unique, culturally specific stressor that Western individualistic models often fail to capture.

### 3.3 Psychophysical monism and the virtue of endurance

Finally, the expression of distress in East Asia is shaped by a cultural history of psychophysical monism, the view that mind and body are inseparable (30). Unlike the Western dualistic tradition that separates mental and physical health, East Asian medical cosmologies (such as Traditional Chinese Medicine) view emotions as inherently somatic. Thus, when a patient presents with “headaches,” “chest tightness,” or “fatigue” rather than sadness, they are not merely using a “strategy” to avoid stigma; they are describing the phenomenological reality of their suffering (31). While this presentation may partly reflect limited emotional literacy, a challenge noted in some clinical observations, it more fundamentally describes the patient’s phenomenological reality where mind and body are experienced as one.

This somatic presentation is reinforced by the cultural virtue of *Ren* (endurance or perseverance). Suffering in silence is frequently valorized as a sign of maturity and strength. A Western clinician trained to value emotional expressiveness may misinterpret this stoicism as “resistance” or “lack of insight.” In reality, the patient is adhering to a cultural script that prioritizes emotional containment and social harmony over individual catharsis (32). Effective prevention models must therefore develop “cultural literacy” to decode these somatic and behavioral idioms of distress, recognizing that in high-context cultures, the body often speaks what the voice cannot.

We can conceptually represent the divergence in suicide risk formation as follows:

The Western Individualistic Risk Model:


$$Risk_{West}\approx f(\text{Internal Psychopathology}+\text{Cognitive Distortion}+\text{Thwarted Belongingness})$$

Intervention Target: The individual cognition and biological symptoms.

The East Asian Relational Risk Model:


$$Risk_{East}\approx f(\text{Moral Shame}+\text{Threat to Collective Face}+\text{Blocked Role Fulfillment})$$

Intervention Target: The interpersonal safety and restoration of social function.

## 4 Toward cultural grounding: implications for practice

It is also worth noting that the binary between Western and Eastern approaches is not absolute. Some Western therapeutic innovations, such as Mindfulness-Based Cognitive Therapy (MBCT) and Acceptance and Commitment Therapy (ACT), have themselves been influenced by Eastern philosophical traditions, representing a form of cross-cultural synthesis that may bridge these divides.

The recognition of these cultural nuances necessitates a move toward “cultural grounding” in suicide prevention (see **Table 1**). This does not imply discarding established Western models, but rather enriching them with emic (insider) perspectives to ensure they are responsive to local realities. We propose several pathways for integrating cultural sensitivity into clinical practice and public health strategies.

**Table 1:** Proposed shifts from standard Western CBT to culturally grounded interventions in East Asian contexts.

Standard western approach	Culturally grounded adaptation
**Cognitive Restructuring: Challenge** “I am a burden” as a cognitive distortion (irrational thought).	**Reframing & Validation:** Validate the desire not to burden others as a virtue (filial piety), then reframe self-care as a method to restore one’s capacity to serve the family long-term.
**Focus:** Individual autonomy and self-actualization.	**Focus:** Relational harmony and restoring social functioning/roles (“Face”).
**Emotion Regulation:** Encouraging direct verbal expression of anger or sadness (Catharsis).	**Action-Oriented Coping:** Utilizing Morita-style acceptance of feelings while focusing on behavioral tasks (e.g., solving the financial debt, resuming studies) to regain honor.
**Confidentiality:** Strict exclusion of family unless imminent risk.	**Conditional Confidentiality:** Early negotiation of a “family safety pact” to involve family as a resource, protecting the patient from isolation.

Bold values represent the primary therapeutic domains or key clinical concepts, while non-bold text provides specific examples of how these concepts are applied in Standard Western CBT versus Culturally Grounded Adaptation.

### 4.1 Reframing the therapeutic narrative

At the individual level, clinicians must navigate the “shame-honor” dynamic with great sensitivity. Standard cognitive techniques that challenge “irrational thoughts” directly may need to be softened. Instead of confronting a patient’s belief that they are a burden, a culturally grounded approach might validate this concern as a sign of their care for the family, while reframing help-seeking as an act of responsibility rather than selfishness. For instance, a therapist might suggest, “By getting support and recovering, you are placing yourself in the best position to fulfill your duties to your family in the long run.” This aligns the intervention with the patient’s values of interdependence and role fulfillment, turning the “burden” narrative on its head. Therapies like Problem-Solving Therapy (PST) and Morita Therapy, which focus on constructive action and accepting emotions (*arugamama*) rather than solely on cognitive restructuring, may be particularly well-suited for patients who prioritize practical competence and emotional containment (33, 34).

### 4.2 Engaging the family as a unit of care

Given the central role of the family in East Asian life, interventions should move from an exclusively individual focus to a family-centered model. While privacy is paramount in Western ethics, in many Asian contexts, excluding the family can be seen as isolating the patient. Effective strategies involve psychoeducation for family members to recognize indirect “idioms of distress,” such as somatization or social withdrawal, which are often precursors to crisis. The “hospital-school-home-community” integrated model currently being explored in parts of China represents a promising step in this direction (35). By creating a “safety net” that includes family and teachers, the burden of seeking help is shared, reducing the individual’s isolation and the risk of “losing face” alone. For clinicians, this necessitates a specific protocol: (1) Conditional Confidentiality: Early in the therapeutic alliance, explicitly negotiate the terms under which family will be involved, framing it as a ‘safety protocol’ rather than a breach of privacy; and (2) Family Psychoeducation: Actively training family members to recognize somatic signs of distress (e.g., changes in sleep or appetite) as equivalent to verbal expressions of suicidal ideation.

It is crucial to note that family-centered approaches must be implemented with sensitivity to the diverse roles families play. While families can be powerful sources of support, they may also be sites of conflict or abuse in some cases. Furthermore, in the aftermath of suicide loss, family members themselves become survivors requiring specialized bereavement support. The ‘family safety net’ approach we advocate must therefore be flexible, trauma-informed, and recognize that for some individuals, chosen support networks outside the biological family may be more appropriate.

### 4.3 Leveraging digital and primary care gateways

To bypass the profound barrier of shame associated with psychiatric clinics, prevention channels must be diversified. Integrating mental health screenings into general medical settings, such as primary care clinics or Traditional Chinese Medicine (TCM) practices, allows individuals to seek help for ‘stress’ or ‘insomnia’ as a culturally acceptable entry point. While increasing mental health literacy remains a long-term goal for the region, utilizing these somatic gateways allows general practitioners to engage at-risk individuals who would otherwise avoid psychiatric labels due to stigma, eventually bridging them toward appropriate mental health interventions. This aligns with the World Health Organization’s Mental Health Gap Action Program (mhGAP), but requires specific training for general practitioners to detect somatic markers of suicide risk (36). Furthermore, anonymous digital platforms offer a critical “face-saving” entry point. Chatbots and online forums allow individuals to disclose suicidal urges without the social risk of being identified, bridging the gap between silent suffering and professional care.

## 5 Future perspectives

Moving forward, the field of suicide prevention must prioritize research that validates culturally specific theoretical models. We need longitudinal studies to determine if constructs like “loss of face” or “interpersonal shame” are more accurate predictors of suicide attempts in East Asian populations than standard Western predictors (37). Additionally, clinical trials comparing standard CBT with culturally adapted versions that incorporate family-oriented modules or indigenous coping strategies are essential to build an empirical evidence base for “culturally grounded” practice.

There is also a need for the development of assessment tools that are sensitive to non-verbal and somatic indicators of risk. Future guidelines for suicide risk assessment in non-Western regions should include inquiries into social shame, family conflict, and somatic distress, alongside standard questions about mood and ideation. Finally, global collaboration should move beyond the unilateral export of knowledge; Western researchers and clinicians have much to learn from the resilience and community-support models found in collectivist cultures, potentially enriching suicide prevention strategies globally.

## 6 Conclusion

The universality of suicide as a tragedy does not imply the universality of its drivers or solutions. While Western-developed models have provided the foundational tools for suicide prevention, their application in East Asian contexts requires deep cultural attunement. By recognizing the power of shame, the importance of “face,” and the primacy of the relational self, we can refine our approaches to be not only scientifically sound but culturally safe. The path forward lies in a synthesis—combining the empirical rigor of evidence-based practice with the wisdom of local cultural realities. This “cultural grounding” is not merely an academic exercise but an ethical imperative to ensure that every individual, regardless of their cultural context, has access to support that understands and respects their lived experience.

## Data Availability

The original contributions presented in the study are included in the article/supplementary material. Further inquiries can be directed to the corresponding author.
